# A Cross-Sectional View on the Comprehensive Multi-Disciplinary Model of Care for a Peri-Natal Mental Health Service Within a Tertiary Women's Hospital in Western Australia

**DOI:** 10.1192/bjo.2024.472

**Published:** 2024-08-01

**Authors:** Chinar Goel, Prisha Goel

**Affiliations:** 1 King Edwards Memorial Hospital, Perth, Australia; 2 The University of Western Australia, Perth, Australia

## Abstract

**Aims:**

King Edward Memorial Hospital (KEMH) is the largest tertiary women's hospital in Western Australia and a tertiary referral center for complex pregnancies, for example, adolescent pregnancies (12–19 yr olds), pregnancies with obstetric complications or fetal anomalies, statewide drug and alcohol antenatal service and preterm births. With 6000 births annually, this women's hospital does not only provide obstetric care, but also looks after gynecology, oncology and chronic pain patients. We would like to share the model of care for our women's mental health service which provides statewide Childbirth and Mental illness (CAMI) service looking after women with chronic enduring mental illness, statewide drug and alcohol antenatal service (WANDAS), adolescent model of care and our service for all other women attending this tertiary hospital within a unique consultation liaison model.

**Methods:**

Our team comprises 3.0 full time consultant psychiatrists, 2 Psychiatry trainee registrars, 5 clinical psychologists, 2 triage nurses and administrative staff. In total, we had 1959 referrals to our service in 2022–23 financial year. These women had varying amount of input from our service during their treatment in hospital: one assessment with advice and signposting to brief therapy, up to a fully comprehensive Multidisciplinary Team (MDT) care as provided by an adult community mental health service. In addition to comprehensive assessment, MDT interventions include risk assessment, pharmacological interventions, psychological interventions, working alongside child protection services, infant mental health and attachment work.

**Results:**

Our most common diagnostic categories included post-traumatic stress disorder (10%), adjustment disorders (10%) followed by Generalized anxiety disorder and recurrent depressive disorder (6% each). Our key performance indicators include: number of consumers (541 in 2022–23) that received comprehensive intervention from us in last 12 mths, consumer and carers’ feedback and rate of completion of outcome scale at point of admission and discharge from service. These figures have remained consistent for the last 5 yrs.

**Conclusion:**

Our hybrid model of care is unique as it incorporates a consultation liaison and a community mental health care model for women attending our hospital. This allows us to provide a safe, specialized, timely service to women in their most vulnerable period of life.

